# Work and everyday life in a digitalized time: Experiences of people with subjective cognitive difficulties related to neurological disorders

**DOI:** 10.1371/journal.pone.0260013

**Published:** 2021-11-15

**Authors:** Monika Lindberg, Maria Ranner, Eva Månsson-Lexell, Lars Jacobsson, Maria Larsson-Lund

**Affiliations:** 1 Department of Health, Education and Technology, Division of Health, Medicine and Rehabilitation, Luleå University of Technology, Luleå, Sweden; 2 Department of Health Sciences, Lund University, Lund, Sweden; 3 Department of Neurology and Rehabilitation Medicine, Skåne University Hospital, Lund-Malmö, Sweden; Universita degli Studi della Campania Luigi Vanvitelli, ITALY

## Abstract

**Introduction:**

Digitalization has changed working life and increased cognitive demands on employees in general. Nevertheless, the consequences for employees with cognitive impairments and subjective cognitive difficulties are to a large extent unexplored. The aim of this study was to explore and describe how employees with subjective cognitive difficulties who are performing digital work tasks experience their vocational situation and how this situation influences their everyday life.

**Methods:**

A qualitative, descriptive, multiple-case study was designed. Self-reports, assessments and qualitative interviews were used to collect data from the seven participants with neurological disorders. The data were analysed using pattern matching.

**Findings:**

The analysed data formed four categories conceptualized as “Working to my full potential”, “Working, but it is largely up to me”, “Working at the expense of everyday life” and “Working without known difficulties”, and these categories included one to four subcategories.

**Conclusion:**

Managing subjective cognitive difficulties in vocational situations and everyday life was challenging in a digitalized working life for participants with neurological disorders. To provide equal access to preventive measures and rehabilitation and a sustainable working life, it is important to investigate the influence of subjective cognitive difficulties systematically on work, everyday life and management strategies in people with neurological disorders in digitalized work.

## Introduction

Digitalization has changed working life and influenced the work environment from physical, organizational and social as well as cognitive points of view. This change implies many new challenges and demands along with opportunities within working life [1–4]. More multitasking, an increased information load and a higher work pace are all examples of consequences of the digitalization of work and workplaces [2, 4, 5]. Many jobs have become borderless, i.e., they can be performed almost independently of time and space, and employees are expected to be flexible, constantly connected, updated and continually learning new aspects related to the digital technology used in their work [2, 4, 6]. These changes have increased the cognitive demands on all employees [2, 4, 5], but these changes can become even more demanding for those experiencing cognitive impairments (CI) [7]. Nevertheless, the consequences of a digitalized working life for employees with CI are to a large extent unexplored in research and often overlooked at workplaces. Hence, it is important to gain more knowledge about how employees with CI experience their work to enhance possibilities for a sustainable working life in a digitalized time.

Approximately 20–30% of Swedish employees have, or are expected to experience, some type of CI during their working life. This impairment can lead to a higher risk of being excluded from the work force [7]. A large proportion of people with neurological disorders such as multiple sclerosis (MS), Parkinson’s disease (PD) or stroke have CIs [8–10]. Previous research confirms that having a neurological disorder and experiencing CI are commonly associated with work-related difficulties or not returning to work [11–14]. The individuals are also less represented in working life and retire at a younger age compared to the general population [8, 12, 15, 16]. CIs in MS, PD and stroke, such as memory and concentration difficulties, sensitivity to disturbing impressions, stress intolerance and fatigue, are barriers for staying or returning to work [12, 17–20]. It is well known that people with neurological disorders can experience cognitive difficulties despite the absence of objective measurable CIs. It is hence important to take into account that CI can be difficult to identify and evaluate [10]. There are many concepts and definitions related to subjective cognitive impairments. The definition of subjective cognitive difficulties (SCDs) used in the present study is recommended by Burmester, Leathem, & Merrick [21]. This definition accounts for difficulties in relation to both memory and other cognitive domains reported by the individual. SCDs are often invisible to others, and this lack of visibility often leads to difficulties not being noticed [18]. To promote a healthy and sustainable working life, employees need to receive the right support at the right time. It is therefore important not only to identify CIs but also SCDs at an early stage.

Even though the demand for cognition has increased in working life, it is important to consider that CIs are one of many factors that affect a person’s ability to work. Work ability is described as relational and affected by the interplay between different dimensions related to personal resources, work, the work environment, and the context, including the wider society and a person’s other activities in life [22–24]. Thus, it is important to take on a broad perspective, focusing on a person’s entire life situation when exploring and supporting a sustainable working life. Support for employees with MS, PD or stroke to stay or return to work consists of interventions at the workplace, adapted work tasks, flexible working schedules [12, 20, 25] and support from colleagues and managers [26, 27]. However, research focusing on how to support employees with CI at work is scarce, particularly in regard to digital work tasks. Moreover, to our knowledge, support related to SCD in working life has received limited attention. To summarize, there is a lack of research about how people with CIs and/or SCDs experience digitalized working life. To promote a healthy and sustainable working life and to reduce exclusion, new knowledge is required to understand the situations they face to improve the design of rehabilitative and preventive measures.

### Aim

The aim of this study was to explore and describe how employees with subjective cognitive difficulties who are performing digital work tasks experience their vocational situation and how this situation influences their everyday life.

## Materials and methods

### Design

A qualitative, descriptive, multiple-case study [28] was chosen. This method is advantageous when exploring the complexity of a person’s current situation in a real context, such as digitalized working life. By combining quantitative and qualitative data, rich in-depth descriptions of each case can be provided (28). This study was approved by the Swedish Ethical Review Authority (Dnr: 2019–06429).

### Participants

Participants were recruited from a database of clients who were discharged from a rehabilitation clinic in northern Sweden from 2018–2020. The selection of potential participants was performed in two steps, because the information in the database did not include sufficient information to determine if potential participants met the inclusion criteria. The inclusion criteria were a) being diagnosed with MS, PD or stroke, b) experiencing SCDs, c) having permanent employment or being self-employed, d) working with digital technology for at least half the working time, e) being able to understand, speak and express themselves in Swedish, and f) living in one of four municipalities near the clinic. For the first step, an occupational therapist (OT) at the clinic screened potential participants in the database for inclusion criteria a, e and f, and the first author (ML) screened for criteria b, c and d for the second step. Those assessed eligible according to the inclusion criteria were then purposefully selected, taking the diagnosis, sex, professions and place of residence into consideration [29]. Potential participants received a letter providing information about the study and were asked to respond to the clinic if they were interested in participating. The letter contained information about participation, including that it was voluntary and the possibility to withdraw from the study at any time without explaining the reason. In cases in which individuals did not respond, the OT contacted them by phone, and those who were interested in participating submitted their contact information to the clinic, which was forwarded to the research team. During the second step, the potential participants were contacted by the first author (ML) for supplementary questions related to inclusion criteria b, c and d about perceived SCDs, type of employment and the number of hours they worked with digital technology during the average workday. In total, twenty-one information letters were sent, and thirteen potential participants showed interest in participating in the study. Of these, three people did not meet inclusion criteria b, c and d. When seven participants had been included, the data were considered sufficiently rich to ensure the quality of the study. Therefore, the remaining three were not screened for inclusion in the second step. Besides the letter, the participants were supplied with oral information before the written informed consent was obtained from all participants. As shown in Table 1, most of the participants were women. Four had a progressive neurological disease while the other three had survived a stroke.

**Table 1:** Characteristics of the participants, n = 7.

*Age*	
median (range)	**51 (32–58)**
*Gender*	
Female	**5**
Male	**2**
*Diagnosis*	
Progressive neurological	**4**
disease (MS₁ = 3, PD₂ = 1)	
Stroke	**3**
*Educational level*	
Upper secondary school	**4**
University	**3**
*Hours at work per day*	
2h	**1**
4h	**5**
8h	**1**
*Occupational group₃*	
Business and Administration	
Professionals	**2**
Business and Administration	
Associate Professionals	**2**
Health Professionals	**1**
Production and Specialized	
Services Managers	**1**
Services and	
Sales Workers	**1**
*Marital status*	
Partner	**2**
Partner, children living at	
home	**3**
Partner, children not living at	
home	**2**

₁ Multiple Sclerosis, ₂ Parkinson’s Disease, ₃ International Labour Organization (ILO), International Standard Classification of Occupations, ISCO-08.

### Data collection and procedure

Data were collected by the first author (ML) through qualitative, open-ended, semi-structured interviews, standardized self-report questionnaires, cognition and work-related assessments with the purpose of obtaining rich, detailed and deep data [28]. The data collection methods were chosen to complement each other to capture both the subjective and objective aspects of how people with SCD experienced their situation. The data were collected during three occasions in the autumn of 2020, and all the sessions with each participant were held within a period lasting a maximum of four weeks.

#### Self-report questionnaires

Before the first data collection session, the participants received four self-report questionnaires by mail to fill out at home. The questionnaires were subsequently collected by the author at the last session at the latest. The first three questions on the self-report Work Ability Index [WAI] [30] were used to assess perceived work ability and resources in relation to work requirements. The first question on the WAI scores ranges from 0 to 10, where 0 stands for “cannot work at all” and 10 stands for “my work ability is at its best”. The second and third questions range from 1 to 5, where 1 stands for “very bad” and 5 stands for “very good”. The Swedish version of Technostress [31, 32] was used to assess how the demands of digital technology affected work and everyday life. In Technostress, 24 items are scored on a five-point ordinal scale ranging from 1 = “strongly disagree” to 5 = “strongly agree”. Occupational Balance Questionnaire 11 [OBQ11] [33] was used to assess experiences of occupational balance in everyday life. The scale ranges from 0 = "completely disagree" to 3 = "completely agree", and the summarized scores range from 0 to 33 points. High scores indicate a higher occupational balance. The Mental Fatigue Scale [MFS] [34] was used to identify the presence of fatigue and how it affected everyday life. The MFS summarized scores range from 0 to 42, and scores over 10.5 points indicate fatigue.

#### Data collection session 1

The first session began with background questions about socio-demographics, work and previously received rehabilitation and vocational rehabilitation. Thereafter, a qualitative open-ended interview [35] was conducted with support from a semi-structured interview guide that included questions about the participants’ vocational situation, experiences of cognitive difficulties at work and how these were managed, support and adaptions at work and whether work affected the participants’ everyday lives. This guide was developed by the authors (ML & MLL) in cooperation with a small discussion group [36] consisting of two stakeholders who met the same inclusion criteria as the participants but were not included in the study. The interviews took place in an environment chosen by the participant, either in person in a setting chosen by the participant (public library n = 1, café n = 1, or first author’s office = 4) or by phone (n = 1) and lasted between 20 and 50 minutes (mean 35 minutes). The interviews were audio recorded and transcribed verbatim, and personal information was omitted to protect the participant´s confidentiality.

#### Data collection session 2

During this session, two standardized assessments using semi-structured interviews were performed. The Work Environment Impact Scale [WEIS] [37, 38] was used to identify how psychosocial and physical factors in the work environment affect satisfaction and well-being at work. The Worker Role Interview [WRI] [39, 40] was used to identify how psychosocial and environmental factors influence participants’ possibilities of remaining at work, returning to work or getting a job. After the WEIS and WRI interviews, the first author (ML) evaluated the answer given on each variable, then scored and summarized them in accordance with the manual. To ensure the quality in evaluating and summarizing the scores, the interviews were recorded as support for the first author (ML) but were not transcribed. The interviews lasted between 30 and 85 minutes (mean 60 minutes) and took place in a location chosen by the participant, either in person at a setting chosen by the participant (public library n = 1, first author’s office n = 4) or by phone (n = 2).

#### Data collection session 3

For this session, the standardized screening instrument, the Montreal Cognitive Assessment [MoCA] [41], was used to assess CIs related to various cognitive functions, such as attention, memory and executive functions. Scores for the MoCA range from 0 to 30, with a score of 26 and higher generally considered normal, implying no CI. The session was conducted as an in-person meeting in an undisturbed setting chosen by the participant (participant´s home n = 2, public library n = 2, or first author’s office n = 3).

### Data analysis

The data were analysed using pattern matching [28]. All the collected data, i.e., open-ended semi-structured interviews, standardized self-report questionnaires and work-related assessments, were read, summarized and merged together as a unit to create a case description for each participant by the first author (ML). In the next step, based on repeated readings of raw data and the case descriptions, the authors (ML and MLL) independently began to search for patterns within each case and across cases. Potential patterns were then compared and discussed jointly, and the analysis continued with identifying similarities and differences between the participant patterns. During this process, it became evident that some participants shared similar experiences of their situation, as reflected in the qualitative interviews and, to some extent, as reflected in the assessments and self-reports. At this stage of the analysis, the elements of the patterns also started to emerge. The participants who had similar patterns, including similar elements, formed a case. In total, the participants formed four different cases, i.e., categories describing the pattern in the different experiences of their vocational situation during digitalized working life. Three cases comprised two participants each, and one comprised only one participant. Each participant was allocated to only one case. All the authors discussed the evolving cases, including their patterns and elements, based on the raw data. In the next step, two of the authors (ML and MLL) continued the analysis by refining each case, their patterns and their elements in a forward-backward procedure between the evolving results and data. Every step taken by one of the authors was discussed with the other to reach agreement. During this process, even if the experiences were divergent, four similar elements were identified across the cases, forming four subcategories in all but one category. Then, all the authors scrutinized, discussed and refined the results to ensure that they were grounded in data and to establish trustworthiness [29]. To ensure the confidentiality of the individual participants in each case/category, their background characteristics are not linked to the four categories in the results.

## Results

The experiences that participants recounted, and the results of the self-report questionnaires and work-related assessments formed four categories (A-D) conceptualized as A) “Working to my full potential”, B) “Working, but it is largely up to me”, C) “Working at the expense of everyday life” and D) “Working without known difficulties”. These categories included one to four subcategories reflecting influence on the vocational situation, influence of social support, adoption of management strategies for occupational balance, and adoption of management strategies and routines at work (Table 2). The findings reveal how SCDs influenced all the participants’ vocational situations and their everyday lives, even though none had measurable mild CIs according to the MoCA (Table 3). All the participants perceived a reduced work ability (Table 3) and some type of technostress caused by work-related demands related to digital technology (Table 4), e.g., an increased workload or constantly changing and evolving technology that put demands on them to learn new things. All the participants in this study had received rehabilitation, but none had specifically received any work-oriented rehabilitation. The categories and subcategories are presented below.

**Table 2:** Categories describing how the participants with subjective cognitive difficulties (n = 7) performing digital work tasks experienced their vocational situation and how the situation influenced their everyday life.

Categories	A) Working to my full potential	B) Working but it is largely up to me	C) Working at the expense of everyday life	D) Working without known difficulties
**Participant**	1 & 2	3 & 4	5 & 6	7
**Subcategories**				
**Influence on vocational situation**	Managing work and being competent despite challenges	Managing work but feeling excluded	Managing work by being flexible	Managing work but reflecting sparsely on the situation
**Influence of social support**	Being supported as needs are made visible	Not being adequately supported at work because needs are not fully understood	Being supported but having unspoken needs that no one identifies	
**Adoption of management strategies for occupational balance**	Prioritizing for an optimal occupational balance	Struggling with occupational balance because of obligations	Loosing occupational balance and valued activities	
**Adoption of management strategies and routines in work**	Using a range of management strategies to enhance work ability	Using management strategies repeatedly to prepare and avoid errors at work	Not using or expressing whether routines and management strategies are needed in work	

**Table 3:** Subjective cognitive difficulties and scores on self-report questionnaires in the four categories of the participants (n = 7).

Category	A	A	B	B	C	C	D
**Participant**	1	2	3	4	5	6	7
*Self-perceived SCD*							
Decreased concentration	Yes	Yes	Yes	Yes	Yes	Yes	Yes
Sensitivity to disturbing stimuli	Yes	Yes	Yes	Yes	Yes	No	Yes
Difficulties with managing several or							
complex tasks	Yes	Yes	Yes	Yes	Yes	Yes	Yes
Memory difficulties	Yes	Yes	Yes	Yes	Yes	Yes	No
Experiencing fatigue	Yes	Yes	Yes	Yes	Yes	No	Yes
*Scores on self-report questionnaires and assessments*							
Fatigue ₁	18.5	19.5	23	22	27.5	12	6.5
Presence of mild cognitive impairment ₂	29	27	27	28	29	27	28
Occupational balance ₃	12	14	13	7	0	24	21
Current work ability compared to when it							
was at its best ₄	5	4	6	6	5	3	8
Current work ability in relation to the							
- physical demands of your work ₅	3	5	3	1	3	4	4
- psychological demands of your work ₆	3	3	3	3	2	3	4

₁ MFS 0–42 points (p), scores >12.5/42 indicates fatigue. ₂ MoCA 0–30 p. scores ≥26/30 normal cognitive state. ₃ OBQ11 0–33 p. ₄ WAI 0–10 p. ₅ WAI 1–5 p. ₆ WAI 1–5 p.

**Table 4:** Rating for each item on Technostress in the four categories of the participants (n = 7).

Category	A	A	B	B	C	C	D
Participant	1	2	3	4	5	6	7
*Techno-overload*							
1. I am forced by this technology to work much faster	1	3	4	3	3	2	4
2. I am forced by this technology to do more work than I can handle	3	4	4	2	4	3	3
3. I am forced by this technology to work with very tight time schedules	4	3	4	1	2	2	3
4. I am forced to change my work habits to adapt to new technologies	2	5	4	5	1	3	3
5. I have a higher workload because of increased technology complexity	3	4	4	3	3	2	2
6. I have to spend a lot of time everyday reading an overwhelming amount of e-mail messages	3	4	2	1	1	1	4
7. I have to work harder because of delays from hardware, software and network problems.	3	2	4	2	2	1	2
*Techno-invasion*							
8. I spend less time with my family due to this technology	1	3	2	4	4	1	3
9. I have to be in touch with my work even during my vacation due to this technology	3	1	1	1	5	1	5
10. I have to sacrifice my vacation and weekend time to keep current on new technologies.	3	1	1	1	5	1	3
11. I feel my personal life is being invaded by this technology	1	5	2	5	5	1	2
*Techno-complexity*							
12. I do not know enough about this technology to handle my job satisfactorily	1	4	3	1	3	3	3
13. I need a long time to understand and use new technologies.	1	4	4	5	3	3	3
14. I do not find enough time to study and upgrade my technology skills.	2	5	4	3	5	2	3
15. I find new recruits to this organization know more about computer technology than I do	1	5	2	1	2	5	3
16. I often find it too complex for me to understand and use new technologies.	1	4	2	2	2	5	3
*Techno-insecurity*							
17. I feel constant threat to my job security due to new technologies	1	3	1	1	1	1	4
18. I am threatened by coworkers with newer technology skills	1	2	1	1	1	1	2
19. I do not share my knowledge with my coworkers for fear of being replaced	1	1	1	1	1	1	2
20. I feel there is less sharing of knowledge among coworkers for fear of being replaced.	2	1	1	1	1	1	3
*Techno-uncertainty*							
21. There are always new developments in the technologies we use in our organization	4	4	5	5	3	3	5
22. There are constant changes in computer software in our organization	3	4	5	3	1	3	4
23. There are constant changes in computer hardware in our organization.	3	2	5	3	1	3	4
24. There are frequent upgrades in computer networks in our organization.	3	2	4	3	1	2	3

Scale ranging from, 1 = “strongly disagree” to, 5 = “strongly agree”.

### Working to my full potential

This category is based on two participants (1 and 2) who both sustained a stroke. Their experiences reflected that despite their SCDs, they were able to work at their present full potential by adopting their own management strategies and support from family, managers and colleagues. They experienced SCDs in all the investigated areas as well as fatigue and a low occupational balance in general (Table 3). The participants described how they were able to continue to have an active everyday life in addition to their work. WEIS and WRI (Tables 5 and 6) supported their work environment and worker role, respectively. They also experienced varied degrees of stress caused by work-related demands related to digital technology according to Technostress (Table 4).

**Table 5:** Rating for each item on The Work Environment Impact Scale (WEIS) in the four categories of the participants (n = 7).

Category	A	A	B	B	C	C	D
Participant	1	2	3	4	5	6	7
1. Time demand	S	S	S	S	I	S	S
2. Task demands	S	S	S	S	I	S	S
3. Appeal of work tasks	S	S	S	S	S	S	S
4. Work schedule	S	S	S	I	I	S	S
5. Co-worker interaction	S	S	S	S	S	S	S
6. Work group membership	S	S	S	S	I	NR	S
7. Supervisor interaction	S	S	I	I	NR	S	S
8. Work role standards	S	S	I	S	S	S	S
9. Work role style	S	S	S	S	S	S	S
10. Interaction with others	S	S	S	S	I	S	S
11. Rewards	S	S	I	I	I	I	S
12. Sensory qualities	S	S	I	I	NR	S	S
13. Physical arrangement	S	S	I	S	NR	S	S
14. Social atmosphere	S	S	I	I	S	S	S
15. Properties of objects	S	S	S	S	I	S	S
16. Physical amenities	S	S	I	S	NR	S	S
17. Meaning of work	S	S	S	S	S	S	S

S = Support, I = Interfere, NR = Not Relevant.

**Table 6:** Rating for each item on The Worker Role Interview (WRI) in the four categories of the participants (n = 7).

Category	A	A	B	B	C	C	D
Participant	1	2	3	4	5	6	7
1. Assesses abilities and limitations	S	S	S	S	S	S	S
2. Expectations of job success	S	S	S	S	I	S	S
3. Takes responsibility	S	S	S	S	S	S	S
4. Commitment to work	S	S	S	S	S	S	S
5. Work-related goals	S	S	S	S	S	S	S
6. Enjoys work	S	S	S	S	S	S	S
7. Pursues interests	S	S	S	I	I	S	S
8. Appraises work expectations	S	S	S	S	S	S	S
9. Influence on other roles	S	S	S	S	S	S	S
10. Work habits	S	S	S	S	S	S	S
11. Daily routines	S	S	S	S	S	S	S
12. Adapts routine to minimize difficulties	S	S	S	S	S	S	S
13. Perception of work setting	S	S	I	I	S	S	S
14. Perception of family and peers	S	S	S	S	I	S	S
15. Perception of boss	S	S	S	S	NR	S	S
16. Perception of co-workers	S	S	S	S	S	S	S

S = Support, I = Interfere, NR = Not Relevant.

#### Managing work and being competent despite challenges

The participants’ experiences showed how they had found ways to work despite being affected by SCDs. They noted that they were aware of how their cognition, a deteriorated memory, sensitivity to stimuli and difficulties when being interrupted, and how they influenced their work ability, saying, *“I also have memory problems […] when I get tired*, *I do not find words*. *And I have to write things down…”* (participant [p] 2). To facilitate their work ability and optimize the working conditions, adaptions were implemented. The participants’ experiences showed that having their own office enabled them to work without interruptions. This resource had a positive effect on their work ability, because it gave them the possibility to structure their work according to their own needs and conditions as described: *“I have worked with this for so long*, *so I have a lot of routine and I think it is still going well…”* (p. 2). As their capacity to work varied from one day to another, they continuously considered how to enhance their work ability. Having flexible working hours and workplaces together with digital solutions made it possible to work remotely if needed and changing the time point for their work as reflected: “*I also have a laptop from work that I have at home so that I can log on if I have to check something or if I do not have the energy to go [to the office] today*. *[*. . .*] I still try to go almost every day*, *I do but if it has been a lot one day*, *then I say that tomorrow I work from home*” (p. 1). Even if the participants noted that their work ability was affected, they still felt competent and found their work meaningful. Due to the awareness of their difficulties, they had lowered the demands on themselves at work but without compromising the quality of their work performance. This capacity was important for them, because they described themselves as being very loyal. Due to their present work ability, they were concerned about not being capable of increasing work hours or changing jobs and having a stagnated future career. Nevertheless, they kept hope for better changes in the future and were grateful for being able to continue to work.

#### Being supported as needs are made visible

The participants indicated that they were open to employers, managers, colleagues and family about their difficulties. This included an openness about their visible, more tangible needs as well as those that were of more invisible character relating to their subjectively perceived cognitive difficulties and fatigue. They observed that by being open, people around them supported them to continue work. Supportive colleagues offered help and support with tasks to reduce the workload or encouraged them to take breaks, as reflected by one participant: *“Yes*, *but now I also get a lot of support through*, *for example*, *my colleague […] So that I do not work too long”* (p.1). The managers were described as supportive in regard to adapting the workload and tasks, working hours and possibilities to take breaks when needed. Having support from families with household chores and lowering their home-related demands also supported their work ability. The participants were grateful for continually being offered support without requesting it and said that this resource enhanced their work ability and enabled a meaningful everyday life.

#### Prioritizing for an optimal occupational balance

These experiences showed how the participants carefully prioritized engagement in activities during everyday life to be able to fulfil their duties at work and thereby uphold important activities in addition to work to achieve occupational balance. They described how their limited capacity and fatigue forced them to make deliberate choices and plan their engagement in activities outside work to maintain their work ability. This prioritization was continuously ongoing, with the aim of finding the best level of engagement over the day and week. The participants also spread out their activities over time in relation to their present desires, capacity and other conditions. An important part of their prioritization was taking time for recovery after work every day. To work, the participants had set many activities outside work “on pause” and abandoned many of their previous activities. To be able to keep up their abilities, they continued to prioritize physical exercise regularly, even though it demanded much of the capacity they had left after work. They also described how they continued doing household chores. Currently, the participants’ leisure activities were fewer, more frequently performed in solitude and consisted mostly of sedentary activities with limited stimuli often performed at home. However, thanks to their priorities and careful planning, they managed to some extent to continue with meaningful social and cultural activities with their friends outside home. This often included deliberate and detailed preparations of the activities and recovery afterwards as reflected: *"I try to do things with friends and meet them and*, *go to a concert but [*..*] I have to rest both before and many times I simply have to take time off the day after"* (p. 2).

#### Using a range of management strategies to enhance work ability

The participants’ experiences showed how they had developed a variety of management strategies, often including a flexible use of technology to prevent or overcome situations that challenged their work ability. As a strategy to minimize stimuli and the risk of being interrupted, they turned off notifications on incoming e-mails and checked them a couple times a day instead. One strategy that was used was described in this way: “… *if you put on a pair of headphones*, *it might look like you’re on the phone or something or sitting and working on something that I do not want to be disturbed in [*..*]*” (p. 1). To support their memory, they used different strategies for reminders, such as writing and checking off to-do lists and using different functions of their mobile phones. Other strategies involved routines to organize different work tasks in an efficient way. For instance, they must be aware of having to continually listen to their varying capacity in relation to their work tasks to find their optimal working situation to support their work ability.

### Working, but it is largely up to me

This category is based on two participants (3 and 4), one who sustained a stroke and one with a progressive neurological disease. Their experiences reflected that their work ability with the presence of SCDs depended much on their own effort to make it work because they felt alone and unsupported by managers and colleagues. Their families were perceived as supporting their possibility to work, but non-working time was influenced by demands and obligations from and to other persons. The participants described how they put the needs of others before their own, despite having memory problems and fatigue that complicated their lives. Their self-reports confirmed SCDs in all areas as well as fatigue and a low occupational balance (Table 3). The WEIS and WRI confirmed that there were several factors in the work environment for both participants that interfered with their performance, satisfaction and well-being at work as well as factors interfering with remaining at work (Tables 5 and 6). The participants experienced varied degrees of technostress in work (Table 4).

#### Managing work but feeling excluded

The participants’ experiences showed that they managed to work despite their SCDs but still felt excluded at work. Their experiences reflected combinations of several cognitively related difficulties that impacted their work ability and worker role, as reflected by saying “[…] *feeling that I am not good enough becomes a reminder that you are not as you used to be*. *And that has*, *it has been difficult in terms of work*. *I have been an*, *I am an incredible ideas man who thinks several steps ahead and not to be able to do it now”* (p. 3). Difficulties with memory and concentration affected the work ability, especially as they often needed to perform several work tasks in parallel or simultaneously. These difficulties also influenced their abilities to compensate for their difficulties, such as when listening to someone and taking notes at the same time, as described, *“[…] it is one thing to sit at a lecture and write words of support for oneself but sit and take notes [at a meeting] and note the decision and feel the uncertainty that I do not have time to write this now*. *I have to do it tomorrow and so I know that tomorrow I may not even understand what I have written” (p*. *3)*. They were aware that stress affected them negatively, both cognitively and physically, and when they were stressed, they were not able to think clearly or in a rational way. It also made them emotional. One participant’s physical ability was affected, and stress negatively affected this ability even more.

The participants said their work had to be performed at the work site, but they had the flexibility to decide when they would work during the day. Learning new things and tasks at the job posed challenges, e.g., learning new technology, even if they indicated that technology foremost facilitated their work. They spoke of adaptations that were agreed upon, such as being given longer time to prepare and perform certain work tasks. However, their capacity varied, and ongoing needs required them to continually manage difficulties to handle their work ability. This made them feel vulnerable in their vocational situation, struggling to manage work on their own. Despite doing the work tasks in a competent way, they expressed a lack of confirmation and communication but also a feeling of not being seen as a fully competent person. Furthermore, because they only had the capacity to perform the job as expected, no more or less, they described losing potential career opportunities.

#### Not being adequately supported at work because needs are not fully understood

The participants experienced a lack of adequate support even if they were open to managers and some of the colleagues about their difficulties and limitations at work. They had requested support from their managers to inform all their colleagues about their difficulties but did not receive support and thereby felt abandoned and unsupported. One participant who also had physical impairments described how difficulties arose when sharing an office with a colleague who was not understanding: *“… I just sit and write and her phone rings and she will be talking*, *then it’s done*. *And then I’ll start carrying my computer out*. *I’ve told her you might be able to go out when you talk*, *but she answers*: *I need the computer…*. *Mmm*, *but [I think] bring your computer [with you] then*, *I may not always have to go away*, *but I have started to do so…*.*”* (p. 3). The participants felt that their needs were overlooked or not fully understood and therefore remained hidden. This concern meant that they repeatedly needed to request support because it was not something that came naturally from others as described: “*…I had to tell them that this is what I should have*. *I have encountered some obstacles*, *and*, *in the beginning*, *it was like*, *the business does not allow that [the support requested]”* (p. 4). They also expressed a feeling of being excluded from the social community and togetherness at work. This was in part related to a sensitivity to auditory stimuli that made it difficult to participate in meetings that many people were attending. Therefore, they chose to take work pauses and lunchbreaks at times and places when it was more peaceful. This in turn affected their sense of inclusion in the workgroup. One expressed the feeling of being excluded from colleagues’ social activities after work, saying, *“No one ever asks me if I want to go to the city [on after work activities]*, *because I walk so slowly*. *It’s so difficult and it’s offensive”* (p. 3).

#### Struggling with occupational balance because of obligations

The participants’ experiences reflected how they struggled with their occupational balance to find equilibrium between capacity and obligations. The participants described how their limited capacity forced them to prioritize and make deliberate choices of which activities to engage in after work so as not to impact their work ability the next day. Activities outside work were routine-based and consisted of mostly similar activities every day. To cope with home-related demands, they needed to rest after work to recover. Their prioritization of activities depended on obligations to close persons with extensive needs rather than their own needs. The participants indicated that these home-related demands were ongoing and woven into their everyday lives and had to be addressed regardless of their capacity or other conditions, as reflected *“*. . .*then it [the obligations and demands to others] keeps going on all the time*, *so I always have the thought that next week*, *then I will do nothing”* (p.4). In addition to these activities, household chores had to be performed, which restricted their engagement in leisure activities. In the current situation, their leisure activities consisted of sedentary activities with limited stimuli and were mostly performed at home. Social events and other activities outside the home were experienced as too demanding and were therefore seldom a choice, even if they expressed missing them.

#### Using management strategies repeatedly to prepare and avoid errors at work

The participants described how they had developed routines and strategies that they used repeatedly to avoid stressful situations and errors as well as to support their work ability as described, “*And there I also try to have a certain strategy that I try to check what is important to me”* (p. 4). Because they were worried about forgetting things or making mistakes, they had adapted different memory-related management strategies that included the use of technology such as leaving reminders on their mobile phones. They also spoke of routinely reading information multiple times before performing a task. Another routine was carefully planning the next working day in advance and ticking off a list with work tasks when they were done. They expressed that when they used management strategies and had control over the day, they minimized the risk of ending up in stressful situations and making mistakes.

### Working at the expense of everyday life

This category includes two participants (5 and 6) with a progressive neurological disease. They described that working was possible mostly because they were flexible and worked when they had the capacity to do so. Their non-working time was almost non-existent because of their prioritization, and borderless work meant that they spent a great deal of time recovering from and preparing for work. The participants described having support from family, managers and colleagues. Additionally, they wanted more support but had difficulties expressing what their needs were. The participants described being affected by deteriorated memory and difficulties concentrating. One participant reported SCDs in all areas, fatigue and a low occupational balance, while the other participant did not report SCDs in all areas or fatigue and experienced an occupational balance (Table 3). The WEIS scores showed that one participant was supported by the work environment, but other factors interfered with the other (Table 5). Overall, the WRI scores showed that both participants had support in their possibilities of remaining at work (Table 6), and Technostress showed that they experienced varied degrees of stress caused by digital technology at work (Table 4).

#### Managing work by being flexible

The participants described how they managed to continue to work despite their SCDs because of flexibility. Their experiences showed how both their physical impairments and cognitive difficulties reduced their work ability. A deteriorated memory impacted their work ability as well as everyday life as reflected, *“… but what I notice clearly now lately is when someone has said something the week before*, *it it’s like blown away*, *I have not heard it*, *it does not remain in the brain at all*. *It’s just blown away”* (p. 6). Cognitively challenging tasks that required focus and concentration were described as tiresome and affecting their work ability. Physical aspects such as difficulties walking and deteriorating function in the upper limbs also influenced their work ability, e.g., the ability to use a computer. Work was described as demanding all the capacity that the participants had, and because their capacity varied, the participants were able to work only when they could. This characteristic led to borderless work that often extended to evenings and weekends, with no fixed times when it started or ended as described: *“I do what I can and then I take a break*, *coffee break and so on*, *I try to go on for a while and so on*, *then I have to compensate for it in the mornings or later or in the evening…”* (p. 6). Participants described working more hours than their work actually required to get the work done. They expressed a fear of an even more deteriorated work ability and of losing their jobs. Nevertheless, their work was an important part of an independent everyday life.

#### Being supported but having unspoken needs that no one identifies

The participants’ experiences reflected that they received substantial support, but they had a feeling of having needs that could not be expressed. Their experiences reflected that their difficulties were obvious, and they were open about their decreased work ability and were supported by employers, managers, colleagues and family. This support was far-reaching when it came to flexibility in time and place of work to enable their continued work. Both described how they were part of a group of colleagues who were aware of and mutually supported each other’s competences by helping each other out with tasks related to, e.g., digital technology and digital connections. Even though receiving a great deal of support, they expressed a feeling of still having needs that neither themselves nor their managers or colleagues could identify. As described in this quotation, “*They [colleagues and managers] are there for me in all possible ways […] The manager has also said*:*—Tell us if you need anything or if we should buy some aids*, *we will buy it at once […] but I’m very bad at getting help both at home*, *at work*, *from occupational therapist and so on*” (p. 6).

#### Losing occupational balance and valued activities

The participants’ experiences reflected how their prioritization of work caused them to lose occupational balance and other valued activities in everyday life. The participants described having limited energy to perform activities in addition to work, and their need for rest and recovery was extensive. The work took almost all their capacity, and they therefore had limited possibilities to choose or prioritize other activities. Their non-working time, which was flexible, was interfered with by constantly being available for work through e-mail or phone after working hours. Therefore, they made many sacrifices to their free time to be able to work. The activities performed besides work were reflected as necessary tasks that had to be done, and the enjoyment of engagement seemed to be lost as reflected, *“I feel like I ’ve forgotten how it is to have fun*. *[*. . .*] I’m just focused on getting things done and ticked off*, *it’s like that’s it […] I just want to get rid of everything so I can take it easy*. *It’s like the main focus…”* (p. 5). They described taking part in household chores to a limited extent and expressed a feeling of being a burden to their close persons. The participants expressed that in addition to work, they were almost never engaged in activities outside the home, with the exception of one of the participants who struggled with upholding physical training routines. Their experiences reflected that the capacity that they had left only allowed them to engage in sedentary quiet activities, often performed alone, such as watching television or browsing the internet.

#### Not using or expressing whether routines and management strategies are needed in work

The participants’ descriptions reflected a sparse use of management strategies or routines to support their work ability or activities in everyday life. They had difficulties identifying specific actions or tasks at work that were challenging. Deteriorated memory was experienced, but these participants did not have any routinely used strategies for how to cope with it. They made written lists or left piles of paper visible as a reminder of things to do, but this did not always help them. They noted the possibilities of using technology as a memory aid but reflected that it demanded that they remember to use it the way intended as reflected: *“…yes*, *you can do that [use the mobile phone for reminders]*, *some things you can write down as memoirs*, *[…] but it is important to remember to write those as well”* (p. 6). They reflected on their lack of management strategies and the potential that they might have to facilitate work and everyday life, but simultaneously, they also described a limited interest in making use of such strategies. One spoke of the need for support from professionals to solve challenges in life and obtain an occupational balance.

### Working without known difficulties

This category is based on the experiences of one participant (7) with a progressive neurological disease. The participant had SCDs and difficulties reflecting and describing the vocational situation during the interviews. No reflections were made in the interviews about whether their work ability had changed after being diagnosed or if it was the same as it had always been. The self-report on WAI showed a present capacity lower than when it was at its best (Table 3). The participant expressed having fatigue that was not confirmed by the self-report but experienced having an occupational balance in everyday life (Table 3). According to the WEIS and WRI (Tables 5 and 6), the participants’ work environment and worker role were supported. The participant experienced technostress (Table 4).

#### Managing work but reflecting sparsely on the situation

Participant seven experienced SCDs and told of being open with the diagnosis with family, managers and colleagues at the same time that the consequences of the SCDs and the diagnosis were reflected upon sparsely. Although the participant described the SCDs in various forms in the screening, the participant was not able to describe them or whether they affected their work ability, work environment, worker role or everyday life. The participant described the vocational situation as satisfactory, and the work was performed as before without any adaptations. If adaptations or support are needed in the future, it will be available. Despite feeling fatigued sometimes at work, the participant did not describe it as something that affected their ability to work, saying *“I am probably more tired now than before*. *[*..*]*, *it is not like that every day [*..*] but you can feel when you get home that you are pretty or a bit tired*, *but you recover quickly”* (p. 7). On direct questions, the participant said that being interrupted when performing a work task was somewhat disturbing, including receiving e-mail notifications, but did not affect their work ability. During the interviews, the participant answered questions shortly with limited reflection. On repeated occasions, the participant had difficulties answering questions and asked for clarification from the researcher. The participant expressed hope that the disease would not affect the work ability in the future.

## Discussion

The primary results showed how SCDs pose challenges to the vocational situation and everyday life to various extents for the participants with neurological disorders. The extent of the challenges ranged from participants who, despite difficulties, managed to work to their full potential to those who worked under varying degrees and types of difficulties. The results reflect, in relation to theory [7, 22, 42], how cognitive resources and difficulties are influenced by individual situations, including the interaction between the person, the environment, the work and other activities in everyday life. Consequently, when interventions that aim to support the work ability are designed, it is important not only to relate cognition to the person. All the participants experienced SCDs in almost all areas investigated, i.e., concentration, sensitivity to stimuli, memory, multi-tasking and fatigue, and SCDs had in most cases a far-reaching impact on their vocational situation and everyday life. Despite that, no one was found to have mild cognitive impairment according to the cognitive assessment [MoCA] [41] used here. Consequently, there is a risk that cognitive difficulties are overlooked if not short-screening questions about SCD, such as ours (Table 3), as well as a dialogue about more invisible needs are performed. Notably, fatigue was detected in most participants by the MFS [34] implying that also fatigue must be assessed to identify possible invisible needs related to work ability. Even though all participants had SCDs and fatigue and experienced challenges in their work situation, none of them had received any vocational rehabilitation. This implies that it is important not to take work ability for granted in less physically demanding jobs, e.g., digital work in offices, to facilitate a sustainable working life for all employees. To provide equal access to preventive and rehabilitative measures, the results indicate the importance of identifying SCDs and fatigue and their impact on the work situation. However, these results are limited to seven participants and should therefore be interpreted with caution.

Our results indicate the importance of considering the relation between work and other activities in everyday life, because the work abilities of most participants depended on how their activities outside work were managed. This suggests, in accordance with theories [42, 43] that interventions considering a person’s entire activity pattern and subjective experiences of balance between activities of different values as well as demands, are important for a sustainable work ability in people with SCDs [44]. The results ca also be seen as a reflection of the digitalization of the working life [44, 45], that has made work borderless due to the increased flexibility of when, where (time and place) and how work is performed. This is different from previously, when work was seen as a separate activity from other activities in everyday life [44]. These changing demands require that work and other activities be considered at the same time to improve the understanding of how work both influences and is influenced by a borderless everyday life.

Several participants prioritized their work-related obligations at the expense of other activities. Much spare time was spent on recovery for the next workday by performing only the necessary household chores and a few less demanding leisure activities. The positive aspects of working e.g., togetherness and a sense of independence made the participants to work at the edge/margin of their ability. These positive aspects are important for a healthy working life [46], but working at the limit of one´s ability is probably negative for a sustainable working life. This is consistent with research [47], suggesting that the engagement in an activity as work can become a health paradox as it can be both healthy and unhealthy. Based on these results, our study indicates the importance for people with SCDs to receive support in weighing the positive aspects of a healthy working life from a more short-term perspective together with aspects related to a sustainable work life from a more long-term perspective.

The participants had adopted management strategies to prevent or solve problems on their own both at work and in daily activities that were decisive for their possibility to continue working. This finding concurs with previous research [48, 49] showing how people with cognitive impairment use a variety of self-initiated strategies to manage technology use and challenges in daily activities. The results indicate, similar to other research [49], a need to systematically assess each person’s management strategies in vocational rehabilitation. By supporting individuals with SCDs in taking on an active role in adopting (self-) management strategies that function well [49, 50], a sustainable work ability can be facilitated. Because knowledge about management strategies in digitalized working life is limited, more research is needed to prepare rehabilitation practitioners to support people with SCDs.

The participants’ experiences showed how both support and insufficient support from managers and colleagues impacted on their vocational situation. Participants described how they had to request support instead of being offered coherent measures and, also, that adaptations received at work depended greatly upon their own request. This meant that when their needs changed over time, they were only followed up by their initiative. The results indicate a need to develop systematic continuous support and follow-up to enhance the possibilities for a healthy and sustainable working life for persons with SCDs. The results also indicate that when measures were provided, the employees’ individual needs were in focus, and no one told of measures in the working organization/environment such as how it could be brain-friendly [4, 7]. An explanation might be that the increased cognitive demand within the working life is rather new [7]. Another explanation might be that SCDs, and fatigue are invisible, stigmatizing and seldom discussed [10, 18]. More research is needed about employers’ perspectives of the increased cognitive demand in the work environment and how preventive measures can be implemented. This knowledge can support employers as well as rehabilitation practitioners when employees with SCDs interact with managers and colleagues.

When the self-reports, assessments and interviews for each participant were scrutinized and compared, gaps were revealed in some persons. For instance, participant 5 had a high occupational balance, but the opposite was found during the interview, whereas participant 7 had difficulties describing the work situation during the interview, but the difficulties were reflected to a limited extent in the assessments. Overall, commonly used instruments for assessing the work environment and the worker role showed few areas that interfered with their work. Instead, the use of the Occupational balance questionnaire and Technostress added important perspectives. Additionally, assessments other than those used in this study might add valuable information. Previous research emphasizes the need to combine self-report questionnaires with observations to explore the use of technology at work [51, 52]. Taken together, this indicates that a combination of various types of data collection methods focusing on different aspects are needed to identify the needs people with SCDs may have in relation to their digitalized working life. Thus, to promote a healthy and sustainable working life, the discussions above indicate the need of considering whether assessments used provide sufficient support for a person-centred intervention for people with SCDs.

## Methodological considerations

Due to the qualitative design of the study, the findings cannot be generalised, but they may be transferable [29] to other people in a similar situation. The combination of various data collection methods resulted in rich and comprehensive description of each of the four cases, that enhance credibility of the findings and provide opportunities for assessment of the transferability. As it was beyond the scope of this qualitative study to focus on difference in the working situation related to background data such as gender, age, diagnosis, educational level, occupational groups and hours at work per day, future research with a quantitative design is needed. Also, due to ethical reasons it was only possible to link diagnosis groups (of all background data) to the cases to ensure the confidentiality of the participants. With respect to the borderless everyday life and the knowledge that gender influence both work- and home-related demands as well as work related health [53], gender can be of particular interest to consider in future research of the digitalised working life. Moreover, as depression is frequent in the studied sample [54] and can influence cognition [55], this is an additional factor that can be considered in relation to cognition in future research. However, in this study no signs of depression were noted in the participants during the three data collection sessions. To enhance the credibility and close the gap between researcher and stakeholders [36], the interview guide was developed in cooperation with two persons who meet the same inclusion criteria as the sample. The authors various experience of rehabilitation for the investigated groups as well as experience in using qualitative methods strengthen the integration of different perspectives during analysis. To ensure the trustworthiness of the analysis [29] the authors took on different roles to scrutinize and analyse data to refine the results and ensure that it was grounded in data.

## Conclusion

SCD challenges the vocational situation and everyday life to various extents for participants with neurological disorders. It is therefore important to investigate their overall activity pattern and occupational balance systematically to provide for sustainable working and everyday life.
