# Supplementary material for: Work and everyday life in a digitalized time: Experiences of people with subjective cognitive difficulties related to neurological disorders
Source: PLoS One. 2021 Nov 15;16(11):e0260013. doi: 10.1371/journal.pone.0260013 (PMC8592488; doi:10.1371/journal.pone.0260013)
Supplement: Attachment — Submitted filename: Response_to two reviewers_210922.docx [file pone.0260013.s001.docx]

Dear reviewers,

Thank you very much for the valuable and constructive comments manuscript entitled “*Work and everyday life in a digitalized time: experiences of people with subjective cognitive difficulties related to neurological disorders*” (PONE-D-21-21244), which helped us develop the manuscript. We have carefully considered all comments and made appropriate changes throughout the manuscript. The revisions are seen in the attached manuscript by the function “Track Changes” and an unmarked version is also attached.

Sincerely,

Monika Lindberg, Maria Ranner, Eva Månsson Lexell, Lars Jacobsson and Maria Larsson Lund

**Reviewers Comments to Author:**

Reviewer #1:

1) Authors should report and present demographic and clinical characteristics of the patients in more detail in the results section. Might be relevant to discuss how not only gender but also age, occupational group, living situation, hours at work per day etc. could have influenced the results. In this line, different neurological disease could have affected the vocational situation of patients who are performing digital work tasks. Therefore, could be interesting to know the distribution of MS, Parkinson and stroke in the different pattern groups. Authors should also discuss it.

***Response:*** *As we already mention in the manuscript, we have chosen not to link the background data of the participants due to ethical reasons. We have re-checked our decision and found that it is possible to present groups of diagnosis of the participants in each case in the results section. However, it is not possible to link to the age, gender, vocational status and hours of working per day to the cases in the results. The seven participants in the study live in a small local area and by adding more background details to each case, we would not be able to protect their anonymity. This can be seen as a limitation, but the aim of qualitative studies is not to study relationships and draw conclusions based on background variables. Future quantitative research with larger samples will be more appropriate to take on such research questions. We have now clarified this reasoning in the methodological considerations.*

2) Depression is a frequent manifestation of stroke, MS and PD and could independently challenges how patients with neurological disease experience their vocational situation in a “digitalized environment”. Therefore, patients should be screened for the presence of depression. The lack of this information is a relevant limit in my opinion and should be, at least, mentioned and discussed.

***Response:*** *Depression was not investigated in the sample. However, the data collector has long clinical experiences of meeting individuals with diagnosis as in the current sample and did not notice any sign of depression during the three data collections sessions with the seven participants. The data collection included many instruments, and we made deliberate choices of which one that were most important to use, to reduce the load on the participants. We have now added a few sentences about the importance of investigating depression in clinical practice and future research in the methodological discussion.*

3) “This finding can be explained by the fact that the MoCA was not developed to identify CIs in people performing digital work tasks.” I don’t understand this sentence…

***Response:*** *This sentence was unclear and not needed in the manuscript. The sentence has been removed from the manuscript.*

Reviewer #2:

Despite the very low of participant in the study, this work addresses to understand how cognitive resources and difficulties are influenced by individual situations, as already demonstrated, considering the interaction between the person, the environment, the work and other activities in everyday life. The issue of quality of life is actually hardly wanted, so this study can add a new gusset. I only suggest the authors to reduce Discussion and Methodological considerations in length and add in methods if patients appreciate to be involved in this study and eventually why.

***Response:*** *The discussion and methodological considerations sections are reduced in length with about a page. All participants agreed to voluntary participate in the study. They gave their informed consent both verbally and in written form before their participation. This is now more clearly specified in the manuscript (Method section). We did not specifically ask if they appreciated to be involved in this study and can therefore not add that in the manuscript. However, the results reflect that they found the research, both the interview questions as well as the assessments relevant and important in relation to their situation.*
