# Supplementary material for: Work and everyday life in a digitalized time: Experiences of people with subjective cognitive difficulties related to neurological disorders
Source: PLoS One. 2021 Nov 15;16(11):e0260013. doi: 10.1371/journal.pone.0260013 (PMC8592488; doi:10.1371/journal.pone.0260013)
Supplement: Peer review history

# Peer review: Decision Letter 0

**DOI:** 10.1371/journal.pone.0260013.r001

**Authors:** Luigi Lavorgna

10 Aug 2021

PONE-D-21-21244

Work and everyday life in a digitalized time: experiences of people with subjective cognitive difficulties related to neurological disorders

PLOS ONE

Dear Dr. Lindberg,

Thank you for submitting your manuscript to PLOS ONE. After careful consideration, we feel that it has merit but does not fully meet PLOS ONE’s publication criteria as it currently stands. Therefore, we invite you to submit a revised version of the manuscript that addresses the points raised during the review process.

Please submit your revised manuscript by Sep 24 2021 11:59PM. If you will need more time than this to complete your revisions, please reply to this message or contact the journal office at plosone@plos.org. When you're ready to submit your revision, log on to https://www.editorialmanager.com/pone/ and select the 'Submissions Needing Revision' folder to locate your manuscript file.

Please include the following items when submitting your revised manuscript:

- A rebuttal letter that responds to each point raised by the academic editor and reviewer(s). You should upload this letter as a separate file labeled 'Response to Reviewers'.
- A marked-up copy of your manuscript that highlights changes made to the original version. You should upload this as a separate file labeled 'Revised Manuscript with Track Changes'.
- An unmarked version of your revised paper without tracked changes. You should upload this as a separate file labeled 'Manuscript'.

If you would like to make changes to your financial disclosure, please include your updated statement in your cover letter. Guidelines for resubmitting your figure files are available below the reviewer comments at the end of this letter.

If applicable, we recommend that you deposit your laboratory protocols in protocols.io to enhance the reproducibility of your results. Protocols.io assigns your protocol its own identifier (DOI) so that it can be cited independently in the future. For instructions see: http://journals.plos.org/plosone/s/submission-guidelines#loc-laboratory-protocols. Additionally, PLOS ONE offers an option for publishing peer-reviewed Lab Protocol articles, which describe protocols hosted on protocols.io. Read more information on sharing protocols at https://plos.org/protocols?utm_medium=editorial-email&utm_source=authorletters&utm_campaign=protocols.

We look forward to receiving your revised manuscript.

Kind regards,

Luigi Lavorgna

Academic Editor

PLOS ONE

Journal Requirements:

When submitting your revision, we need you to address these additional requirements.

1. Please ensure that your manuscript meets PLOS ONE's style requirements, including those for file naming. The PLOS ONE style templates can be found at

https://journals.plos.org/plosone/s/file?id=wjVg/PLOSOne_formatting_sample_main_body.pdf and

https://journals.plos.org/plosone/s/file?id=ba62/PLOSOne_formatting_sample_title_authors_affiliations.pdf

2. Please provide additional details regarding participant consent. In the Methods section, please ensure that you have specified (1) whether consent was informed and (2) what type you obtained (for instance, written or verbal). If your study included minors, state whether you obtained consent from parents or guardians. If the need for consent was waived by the ethics committee, please include this information.

3. In your Data Availability statement, you have not specified where the minimal data set underlying the results described in your manuscript can be found. PLOS defines a study's minimal data set as the underlying data used to reach the conclusions drawn in the manuscript and any additional data required to replicate the reported study findings in their entirety. All PLOS journals require that the minimal data set be made fully available. For more information about our data policy, please see http://journals.plos.org/plosone/s/data-availability.

Upon re-submitting your revised manuscript, please upload your study’s minimal underlying data set as either Supporting Information files or to a stable, public repository and include the relevant URLs, DOIs, or accession numbers within your revised cover letter. For a list of acceptable repositories, please see http://journals.plos.org/plosone/s/data-availability#loc-recommended-repositories. Any potentially identifying patient information must be fully anonymized.

Important: If there are ethical or legal restrictions to sharing your data publicly, please explain these restrictions in detail. Please see our guidelines for more information on what we consider unacceptable restrictions to publicly sharing data: http://journals.plos.org/plosone/s/data-availability#loc-unacceptable-data-access-restrictions. Note that it is not acceptable for the authors to be the sole named individuals responsible for ensuring data access.

We will update your Data Availability statement to reflect the information you provide in your cover letter.

[Note: HTML markup is below. Please do not edit.]

Reviewers' comments:

Reviewer's Responses to Questions

**Comments to the Author**

1. Is the manuscript technically sound, and do the data support the conclusions?

The manuscript must describe a technically sound piece of scientific research with data that supports the conclusions. Experiments must have been conducted rigorously, with appropriate controls, replication, and sample sizes. The conclusions must be drawn appropriately based on the data presented. 

Reviewer #1: Partly

Reviewer #2: Yes

**********

2. Has the statistical analysis been performed appropriately and rigorously? 

Reviewer #1: N/A

Reviewer #2: Yes

**********

3. Have the authors made all data underlying the findings in their manuscript fully available?

The PLOS Data policy requires authors to make all data underlying the findings described in their manuscript fully available without restriction, with rare exception (please refer to the Data Availability Statement in the manuscript PDF file). The data should be provided as part of the manuscript or its supporting information, or deposited to a public repository. For example, in addition to summary statistics, the data points behind means, medians and variance measures should be available. If there are restrictions on publicly sharing data—e.g. participant privacy or use of data from a third party—those must be specified.

Reviewer #1: Yes

Reviewer #2: Yes

**********

4. Is the manuscript presented in an intelligible fashion and written in standard English?

PLOS ONE does not copyedit accepted manuscripts, so the language in submitted articles must be clear, correct, and unambiguous. Any typographical or grammatical errors should be corrected at revision, so please note any specific errors here.

Reviewer #1: Yes

Reviewer #2: Yes

**********

5. Review Comments to the Author

Please use the space provided to explain your answers to the questions above. You may also include additional comments for the author, including concerns about dual publication, research ethics, or publication ethics. (Please upload your review as an attachment if it exceeds 20,000 characters)

Reviewer #1: The article of Lindberg and colleagues explored describe how employees with subjective cognitive

difficulties who are performing digital work tasks experience their vocational situation

and how this situation influences their everyday life. The topic is really interesting but this study is only a description of seven cases and therefore the relevance of the results is only partial....

Some major concerns:

1) Authors should report and present demographic and clinical characteristics of the patients in more detail in the results section. Might be relevant to discuss how not only gender but also age, occupational group, living situation, hours at work per day etc. could have influenced the results. In this line, different neurological disease could have affected the vocational situation of patients who are performing digital work tasks. Therefore, could be interesting to know the distribution of MS, Parkinson and stroke in the different pattern groups. Authors should also discuss it.

2) Depression is a frequent manifestation of stroke, MS and PD and could independently challenges how patients with neurological disease experience their vocational situation in a “digitalized environment”. Therefore, patients should be screened for the presence of depression. The lack of this information is a relevant limit in my opinion and should be, at least, mentioned and discussed.

3) “This finding can be explained by the fact that the MoCA was not developed to identify CIs in people performing digital work tasks.” I don’t understand this sentence…

Reviewer #2: Despite the very low of partecipante in the study, this work addresses to understand how cognitive resources and difficulties are influenced by individual situations, as already demonstrated, considering the interaction between the person, the environment, the work and other activities in everyday life. The issue of quality of life is actually hardly wanted, so this study can add a new gusset. I only suggest the authors to reduce Discussion and Methodological considerations in length, and add in methods if patients appreciate to be involved in this study and eventually why.

**********

6. PLOS authors have the option to publish the peer review history of their article (what does this mean?). If published, this will include your full peer review and any attached files.

If you choose “no”, your identity will remain anonymous but your review may still be made public.

**Do you want your identity to be public for this peer review?** For information about this choice, including consent withdrawal, please see our Privacy Policy.

Reviewer #1: No

Reviewer #2: No

[NOTE: If reviewer comments were submitted as an attachment file, they will be attached to this email and accessible via the submission site. Please log into your account, locate the manuscript record, and check for the action link "View Attachments". If this link does not appear, there are no attachment files.]

While revising your submission, please upload your figure files to the Preflight Analysis and Conversion Engine (PACE) digital diagnostic tool, https://pacev2.apexcovantage.com/. PACE helps ensure that figures meet PLOS requirements. To use PACE, you must first register as a user. Registration is free. Then, login and navigate to the UPLOAD tab, where you will find detailed instructions on how to use the tool. If you encounter any issues or have any questions when using PACE, please email PLOS at figures@plos.org. Please note that Supporting Information files do not need this step.

# Author comment: Author response to Decision Letter 0

**DOI:** 10.1371/journal.pone.0260013.r002

19 Oct 2021

Dear reviewers,

Thank you very much for the valuable and constructive comments manuscript entitled “Work and everyday life in a digitalized time: experiences of people with subjective cognitive difficulties related to neurological disorders” (PONE-D-21-21244), which helped us develop the manuscript. We have carefully considered all comments and made appropriate changes throughout the manuscript. The revisions are seen in the attached manuscript by the function “Track Changes” and an unmarked version is also attached.

Sincerely,

Monika Lindberg, Maria Ranner, Eva Månsson Lexell, Lars Jacobsson and Maria Larsson Lund

Reviewers Comments to Author:

Reviewer #1:

1) Authors should report and present demographic and clinical characteristics of the patients in more detail in the results section. Might be relevant to discuss how not only gender but also age, occupational group, living situation, hours at work per day etc. could have influenced the results. In this line, different neurological disease could have affected the vocational situation of patients who are performing digital work tasks. Therefore, could be interesting to know the distribution of MS, Parkinson and stroke in the different pattern groups. Authors should also discuss it.

Response: As we already mention in the manuscript, we have chosen not to link the background data of the participants due to ethical reasons. We have re-checked our decision and found that it is possible to present groups of diagnosis of the participants in each case in the results section. However, it is not possible to link to the age, gender, vocational status and hours of working per day to the cases in the results. The seven participants in the study live in a small local area and by adding more background details to each case, we would not be able to protect their anonymity. This can be seen as a limitation, but the aim of qualitative studies is not to study relationships and draw conclusions based on background variables. Future quantitative research with larger samples will be more appropriate to take on such research questions. We have now clarified this reasoning in the methodological considerations.

2) Depression is a frequent manifestation of stroke, MS and PD and could independently challenges how patients with neurological disease experience their vocational situation in a “digitalized environment”. Therefore, patients should be screened for the presence of depression. The lack of this information is a relevant limit in my opinion and should be, at least, mentioned and discussed.

Response: Depression was not investigated in the sample. However, the data collector has long clinical experiences of meeting individuals with diagnosis as in the current sample and did not notice any sign of depression during the three data collections sessions with the seven participants. The data collection included many instruments, and we made deliberate choices of which one that were most important to use, to reduce the load on the participants. We have now added a few sentences about the importance of investigating depression in clinical practice and future research in the methodological discussion.

3) “This finding can be explained by the fact that the MoCA was not developed to identify CIs in people performing digital work tasks.” I don’t understand this sentence…

Response: This sentence was unclear and not needed in the manuscript. The sentence has been removed from the manuscript. 

Reviewer #2:

Despite the very low of participant in the study, this work addresses to understand how cognitive resources and difficulties are influenced by individual situations, as already demonstrated, considering the interaction between the person, the environment, the work and other activities in everyday life. The issue of quality of life is actually hardly wanted, so this study can add a new gusset. I only suggest the authors to reduce Discussion and Methodological considerations in length and add in methods if patients appreciate to be involved in this study and eventually why.

Response: The discussion and methodological considerations sections are reduced in length with about a page. All participants agreed to voluntary participate in the study. They gave their informed consent both verbally and in written form before their participation. This is now more clearly specified in the manuscript (Method section). We did not specifically ask if they appreciated to be involved in this study and can therefore not add that in the manuscript. However, the results reflect that they found the research, both the interview questions as well as the assessments relevant and important in relation to their situation.

Attachment Submitted filename: Response_to two reviewers_210922.docx Click here for additional data file.

# Editor report: Decision Letter 1

**DOI:** 10.1371/journal.pone.0260013.r003

**Authors:** Luigi Lavorgna

2 Nov 2021

Work and everyday life in a digitalized time: experiences of people with subjective cognitive difficulties related to neurological disorders

PONE-D-21-21244R1

We’re pleased to inform you that your manuscript has been judged scientifically suitable for publication and will be formally accepted for publication once it meets all outstanding technical requirements.

Within one week, you’ll receive an e-mail detailing the required amendments. When these have been addressed, you’ll receive a formal acceptance letter and your manuscript will be scheduled for publication.

An invoice for payment will follow shortly after the formal acceptance. To ensure an efficient process, please log into Editorial Manager at http://www.editorialmanager.com/pone/, click the 'Update My Information' link at the top of the page, and double check that your user information is up-to-date. If you have any billing related questions, please contact our Author Billing department directly at authorbilling@plos.org.

If your institution or institutions have a press office, please notify them about your upcoming paper to help maximize its impact. If they’ll be preparing press materials, please inform our press team as soon as possible -- no later than 48 hours after receiving the formal acceptance. Your manuscript will remain under strict press embargo until 2 pm Eastern Time on the date of publication. For more information, please contact onepress@plos.org.

Kind regards,

Luigi Lavorgna

Academic Editor

PLOS ONE

Additional Editor Comments (optional):

Reviewers' comments:

# Editor report: Acceptance letter

**DOI:** 10.1371/journal.pone.0260013.r004

**Authors:** Luigi Lavorgna

4 Nov 2021

PONE-D-21-21244R1 

Work and everyday life in a digitalized time: experiences of people with subjective cognitive difficulties related to neurological disorders 

Dear Dr. Lindberg:

I'm pleased to inform you that your manuscript has been deemed suitable for publication in PLOS ONE. Congratulations! Your manuscript is now with our production department. 

If your institution or institutions have a press office, please let them know about your upcoming paper now to help maximize its impact. If they'll be preparing press materials, please inform our press team within the next 48 hours. Your manuscript will remain under strict press embargo until 2 pm Eastern Time on the date of publication. For more information please contact onepress@plos.org.

If we can help with anything else, please email us at plosone@plos.org. 

Thank you for submitting your work to PLOS ONE and supporting open access. 

Kind regards, 

PLOS ONE Editorial Office Staff

on behalf of

Dr. Luigi Lavorgna 

Academic Editor

PLOS ONE
